# A trial of exosome rich stem cell secretome as a potential treatment for Hypermobile Ehlers-Danlos Syndrome

**DOI:** 10.3389/fmed.2026.1945865

**Published:** 2026-09-29

**Authors:** Kevin J. Flynn, John T. Ransom, Daniel McGrath

**Affiliations:** 1 Independent Researcher, Austin, TX, United States; 2 Direct Biologics, Medical Affairs, Austin, TX, United States; 3 McGrath Medical, Austin, TX, United States

**Keywords:** extracellular vesicle, hEDS, human bone marrow mesenchymal stem cell, Hypermobile Ehlers-Danlos syndrome, regenerative therapies, secretome

## Abstract

**Introduction:**

Hypermobile Type Ehlers-Danlos Syndrome (hEDS) accounts for 80%–90% of all Ehlers-Danlos cases. hEDS pathophysiology is uncertain. There is no diagnostic test. Symptoms include joint hypermobility, intractable and debilitating joint pain, chronic fatigue, brain fog, irritable bowel symptoms and mast cell activation syndrome. Treatment options are limited and ineffective. None are disease-modifying. This pilot study evaluated the safety and potential for efficacy of a novel biologic investigational product (IP) comprising an extracellular vesicle enriched secretome from human bone marrow-derived mesenchymal stem cells (hbm-MSC EVs).

**Method:**

Ten (10) hEDS patients were recruited and treated with up to four doses of IP by intravenous infusion for up to five months. Symptoms were assessed using a custom 9-criteria questionnaire addressing common symptoms and by observation at each infusion.

**Results:**

No treatment related adverse events were reported or noted. Statistically significant response was found before treatment and after treatment for the ten patients as a group. However, six (6) out of the ten (10) patient exhibited distinct, substantial, rapid, and sustained overall improvement of their symptoms within the study time frame. Due to time limitations of the study, a single distinct responder (one of six responders), with IND approval for continued treatment, was further treated and observed for an additional twelve (12) months. Treatment was ultimately stopped at the patient's request and for 32 months thereafter has lived in apparent remission without pain or any previous symptoms or signs of Hypermobile Ehlers-Danlos Syndrome. All five (5) prior distinct responders with discontinued treatment reverted to their pre-treatment condition.

**Conclusion:**

Results of this pilot study suggest that a randomized, controlled trial is warranted to evaluate the potential of the IP for treatment of hEDS.

## Introduction

Of the several types of Ehlers-Danlos Syndrome (EDS) the most common type is hypermobile EDS (hEDS) (1–4). With the current US Census estimating approximately 349,000,000 people living in the US and frequency estimates of 1 in 3,100 to 1 in 5,000 according to The Ehlers-Danlos Society, this translates to 70,000 to 112,903 persons afflicted with hEDS in the United States (5).

hEDS appears to be a multisystem disorder with unknown pathophysiology. hEDS inheritance appears to follow an autosomal dominant expression pattern. While genetic bases for other forms of EDS have been defined, diagnosis for hEDS is by clinical means only and there is no genetic or other diagnostic laboratory test (1, 2, 6–12). While many of the other twelve related EDS subtypes have well defined genetic mechanisms, the molecular basis of hEDS has remained undefined and most subtypes are linked to variants of the collagen pathways (1). Recent genomic analysis revealed that hEDS may be linked to multiple genetic variants in i) the adaptive immune system, specifically HLA molecules, ii) the mitochondrial respiratory chain and iii) certain collagen synthesis pathways (13). The most common characteristics are persistently painful hyper-mobile joints, joint dislocations, GI disturbances, headaches, orthostatic tachycardia, fatigue, and brain fog with difficulty concentrating (4, 14, 15). Patients need to address both physical and psychosocial challenges to prevent issues such as unemployment, disrupted educational pathways and dependency (14). It is thought by some that this assembly of symptoms is related in part to Mast Cell Activation Syndrome (MCAS) (16–19). In fact, many symptoms of hEDS, including headaches, peripheral neuropathy, postural orthostatic tachycardia syndrome (POTS), and gastrointestinal, rheumatologic and urogynecologic conditions, are consistent with MCAS (17). While it is also suspected that a disorder in collagen synthesis may play a role in the pathophysiology of hEDS, no specific genetic or other laboratory abnormality has yet been identified. Others have suggested immune system dysfunction may play a role (20, 21).

There are no approved therapeutics or satisfactory treatment options and care is focused on symptom management. Treatment with glycosaminoglycans, NSAIDs, pregabalin, opioids, and other methods offer only temporary and, at best, partial help in ameliorating chronic and debilitating joint pain. HEDS patients also often turn to complementary and alternative medicine treatment options with some success found in massage and nutritional supplements (22). These patients often cannot hold steady jobs, and their life is severely limited although their intellect and life span are not.

Regenerative therapies, such as treatment with stem cells that can promote wound healing and reduce inflammation, may be an appropriate therapeutic approach for hEDS, but there is little literature describing regenerative approaches in any form of EDS. A patient with vascular EDS, which is associated with mutations in COL1A1 and COL3A1 collagen genes, was treated with allogeneic mesenchymal stem cells (MSC) and high dose vitamin C and experienced improved wound healing (23). In the last decade, the MSC secretome, which includes extracellular vesicles (EVs), has been recognized as a new paracrine signaling mechanism that can modify the physiological state of recipient cells (24, 25). Along with growth factor and cytokine signals, EVs are recognized as major agents of cell-to-cell communication due to their ability to directly transfer various biological molecules including messenger RNAs (mRNAs), microRNAs (miRNAs), ligands, fibronectin and receptors from donor cells to recipients. EVs from different cell types (*e.g.,* keratinocytes, adipocytes, endothelial cells, MSC, melanocytes) could bring crucial molecular information to cell-to­cell communication. Bone marrow MSC derived EVs have been shown to regulate and normalize immune responses and reduce inflammation (26–28). The Investigational Product (IP) in this study is a new form of biological therapy that comprises an EV enriched secretome obtained from highly regenerative human bone marrow mesenchymal stem cells (hbm-MSC EVs). The apparent safety, potential for efficacy and potential mechanisms of action (MOA) of hbm-MSC EVs, and of the IP studied here in particular, is the topic of intense study in research literature and active clinical trials. There are likely many MOAs, including anti-inflammatory, immunomodulatory and tissue regenerative capacities, possibly relevant to hEDS (24–36). The IP may additionally contribute to extracellular matrix (ECM) remodeling by delivering various ECM components. Among these are fibronectin (FN1), periostin (POSTN), hyaluronic acid (HA), and several collagens, including types I through VII, XI, XII, XIV, and XVI, which are produced naturally by MSCs.

We hypothesized that the immunomodulatory and regenerative properties of the IP would safely and favorably alter some or all of the common symptoms associated with hEDS (36–39). Here we report the response to treatment of ten hEDS patients using repeated intravenous (IV) administration of the IP.

## Materials and methods

### Ethical considerations

Institutional Review Board (IRB) approval was obtained from the Institute of Regenerative and Cellular Medicine (IRCM-2022-355). Informed Consent was obtained from all patients using the approved signature form approved by the IRB. It was later approved and expanded under IND 29936 to extend treatment duration for one patient.

### Patient selection

Patient demographics included two males and eight females, ranging in age from 21 to 57 years old, and all were consented to participate in the study in accordance with the Institutional Review Board (IRB) and IND procedures. Patients were drawn from a local EDS support group, some of whom contacted other non-local candidates. All had been previously diagnosed with hEDS, usually by multiple consultants and were using various treatments at the start of the study (Table 1). All were clinically interviewed and examined by the PI and found to have physical and historical findings compatible with the diagnosis of hEDS. Participants were instructed to continue using any helpful modality and medication during the study. All but one had seen a rheumatologist to exclude potential autoimmune disease. None were on immunosuppressants. None had a diagnosis of lupus erythematosus although one patient had an Antinuclear Antibody (ANA) test of 360 with no other abnormal blood findings. One patient had prior treatment with extracellular vesicle infusion of placental origin without abatement of symptoms. Two were positive for HLA-B27. One had a mutation in the methylenetetrahydrofolate reductase (MTHFR) gene. Due to the time limitations on the original study approval, an additional approval for extended infusions on a single distinctly responsive patient was obtained for another 12 months. This patient was chosen because of evidence of being a distinct responder and as well as the extensive clinical data available (Tables 2, 3).

**Table 1:** Therapies used by all ten study participants, comorbidities, previous relevant physician visits and other factors prior to their entry into the study. Patient designation as a responder (R) to the IP or non-responder (NR) at the end of the study is also shown in the last column.

**Patient**	**Medications/Therapies**	**Prior Visits**	**Comorbidities**	**Other**	**R/NR**
1	Lyrica, Naprosyn, CBD/THC, Hydrocodone	Rheumatologist		HLA B27+, ANA-	R
2	Advil, massage therapy	Rheumatologist	Interstitial cystitis, possible celiac disease		R
3	Topiramate, Morphine, Oxycodone, Hydrocodone, Advil, Aleve	Pain management			R
4	Flexoril, Aspirin, Kratom, Diamox, Ketamine, Tylenol	Chiropractic			R
5	Norco, Ketamine, Lyrica, Ambien, CBD/THC, Naltrexone, Valium, B vitamins, Mg, Tri-peptides, Cord blood stem cell infusion, physical therapy	Rheumatologist	Light sensitivity, dry eye, salivary inflammation, insomnia	ANA-	NR
6	Methylfolate, THC, Lyrica		Sleep apnea, bilateral hip surgery, anorexia, polycystic ovary syndrome, MTHFR mutation, light sensitivity		NR
7	Gabapentin, Trazadone, Botox, THC/Delta-8, Ibuprofen			ANA-	NR
8	Compazine, Aleve		MCAS, IBS, dysautonomia, light sensitivity	ANA+	NR
9	Celebrex, Advil, Lyrica, gabepentin, PRP injections		POTS, light sensitivity	HLA B27+, ANA-	R
10	Midodrine, ibuprofen, gabapentin	Rheumatologist	POTS, insomnia, gall stones, kidney stones, light sensitivity	ANA-	R

**Table 2:** Extended treatment patient history and work-up.

Patient Symptoms
Headaches including Hangar Headaches.
Brain Fog/Concentration Problems.
Anxiety.
Fatigue.
Chronic, debilitating Joint Pain/Tender Joints worsening at early puberty including bilateral plantar pain.
Joint Subluxations and Dislocations.
Irritable Bowel/Constipation with acute diarrhea.
Mild Asthma.
Lack of Overall Feeling of Well Being.
Patient Signs.
Joint hypermobility at a young age (Beighton Score = 6).
Easily injured with minor trauma.
Joint Subluxations and Dislocations.
High-Arched Palate/Dental Crowding.
Teeth Unresponsive to Orthodontic Treatment.
Physical Flat Feet.
Bicuspid Aortic Valve.
Inability to walk due to plantar and other joint pain.
Normal x-Rays except for mouth/Normal lumbar spine.
Neurology Consultation/Consistent with hEDS.
Geneticist Consultation/Consistent with hEDS.
Rheumatology Consultation/ANA Negative/HLA B27 Positive/No Evidence for SLE, RA or Ankylosing Spondylitis.
Gastroenterology Consultation/Normal upper and lower endoscopy.
Dermatology Consultation/Normal skin tone/No Evidence for Psoriasis/Consistent with hEDS.
Ophthalmology Consultation/Normal eye exam.
Family History.
Patient/Adopted.
Birth Mother/Negative for hEDS.
Birth Father/No available history.

**Table 3:** Therapies used by extended treatment patient prior to entry into the study.

Therapeutic Modality
Physical Therapy
Massage Therapy
NSAIDS
Opioids (Hydrocodone)
Pregabalin (150 mg PO TID)
Gabapentin
THC and CBD

### Investigational product

The IP (DB2Q) is an extracellular vesicle enriched secretome product from human bone marrow-derived mesenchymal stem cells and is currently in Phase III clinical trials for the treatment of acute respiratory distress syndrome (ARDS, NCT05354141). Allogeneic hbm-MSCs are cultured and the secretome is collected, processed, sterilized, and aliquoted into sterile vials according to Current Good Manufacturing Practice (cGMP) protocols and procedures per FDA guidelines to provide product. The IP is manufactured to commercial cGMP standards and subject to strict quality assurance processes that confer all the documented regenerative and immunoregulatory properties of intact hbm-MSCs. The constituent composition and manufacturing of the IP is proprietary but is briefly summarized in the literature (37). The IP contains over 1,000 bioactive molecular species, including miRNAs, other small RNAs, cytokines, chemokines, ECM components, and lipids with regenerative potential (37). The IP was supplied as frozen 5 mL vials. For a single dose three of the 5 mL vials were thawed at room temperature and immediately combined, diluted and gently mixed into 30 mL of normal saline. IP was administered by IV as a single dose volume of 45 mL containing 15 mL of product and repeated in 3 months when symptoms began to return to the six distinct responders in the initial group of ten. Apparent non-responders also continued treatment. When symptoms began to return after another 3 to 4 months additional double doses (24 h apart) were administered in the hope of prolonging preliminary signals of treatment efficacy. Each 45 mL infusion with a total dose of 15 mL of IP contained approximately 1.2 trillion EV particles. The individual dose of 15 mL of IP was indicated by identical safe and effective dosages used in Acute Respiratory Distress Syndrome (ARDS) in several Phase II clinical trials (NCT04493242 and NCT04657458) and an ongoing Phase III RCT (NCT05354141) (32, 35, 37). No severe treatment-related adverse events of any grade according to the Common Terminology Criteria for Adverse Events (CTCAE) have been noted in these or any other clinical investigations across more than 300 patients (29–36, 38–41).

### Protocol

#### Ten patient study

The study was IRB approved on December 21, 2022 and amended October 9, 2023 by the Institute of Regenerative and Cellular Medicine (IRCM-2022-355). And it was approved and expanded under IND 29936. The format was prospective, open label and non-randomized and it was conducted at a single site (Austin, TX). The IP was initially administered by IV as a single dose (total volume of 45 mL containing 15 mL of IP) to ten (10) patients then repeated three to four months later to all ten as symptoms began to return in distinctly responsive patients. The available patient history data, including therapies and comorbidities, is summarized in Table 1. In an attempt to prolong preliminary signals of efficacy, double doses (24 h apart) were also administered. These initial ten patients were treated over five months with a total of 3 to 4 doses (Supplementary Figure. S1). One patient failed to show up for the final dose and for another a vein could not be found for the final infusion.

#### Single patient extended study

To evaluate the safety and efficacy of additional doses on patient responses, an extended dose regimen was then approved for one (1) of the distinctly responsive patients through the FDA's single patient IND process. This additional treatment included 5 more doses, including another double dose, over an additional twelve (12) months (Figure 4). This patient was selected to continue treatment due to the distinct response observed as well as the extensive personal clinical data available (Tables 2, 3).

#### Symptom assessment

Since a validated assessment scoring system specific for hEDS patients was not available at the start of the study, a novel 9-parameter questionnaire using a 10-point scale was developed as a patient reported outcome measure (PROM) to address symptoms commonly associated with hEDS. The questionnaire also included a notes section to capture patient specific information and other clinical notes. Each patient was asked by the investigator to rank symptom severity or medication usage from a scale of 0 (none) to 10 (high) on the following parameters: Headaches; Brain Fog/Concentration; Fatigue; Joint Pain; Joint Looseness/Dislocations; GI Function/Motility; CBD/THC Use; NSAID Use; Overall Feelings of Well Being. The questionnaire shares three symptoms (pain, fatigue, brain fog) with the Ehlers-Danlos directed disability index recently described by Chai et al. after this study commenced (42). Baseline symptoms and symptoms throughout the study were assessed with examination by the PI at each treatment infusion. The questionnaire was administered within 24 h before each dose, within 48 h after each dose and at various intervals between doses and after the final dose. Summed score results across all symptoms for each patient were calculated to generate Figure 1, and results for each individual symptom were compiled from each patient to construct Figure 2.

**Figure 1:**
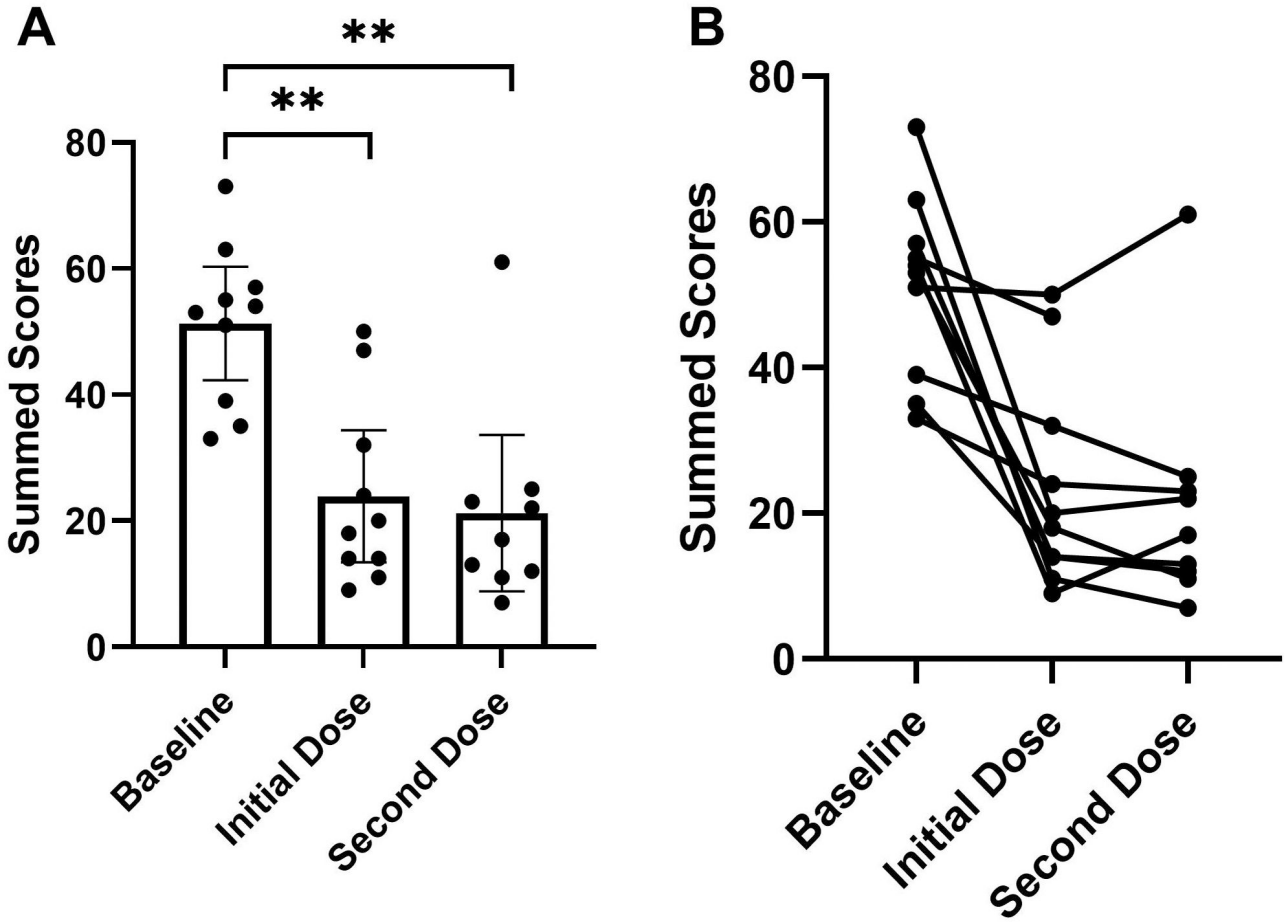
The IP promotes statistically significant and rapid responses in hEDS summed symptom scores across the entire patient group. Initial and second doses of the IP were provided as described in the Protocol section of Materials and Methods. **(A)** All symptom scores for each patient were recorded prior to IP infusions (baseline) and after the initial and second dose regimens. Plots are shown with dot symbols representing individual patients, box height indicating the mean and whiskers showing 95% confidence intervals. Comparisons (t-test) were performed between baseline scores and those following each dose regimen; **p < 0.01. **(B)** Individual patient scores obtained across the dosing protocol are shown as continuous lines and data points for each patient.

**Figure 2:**
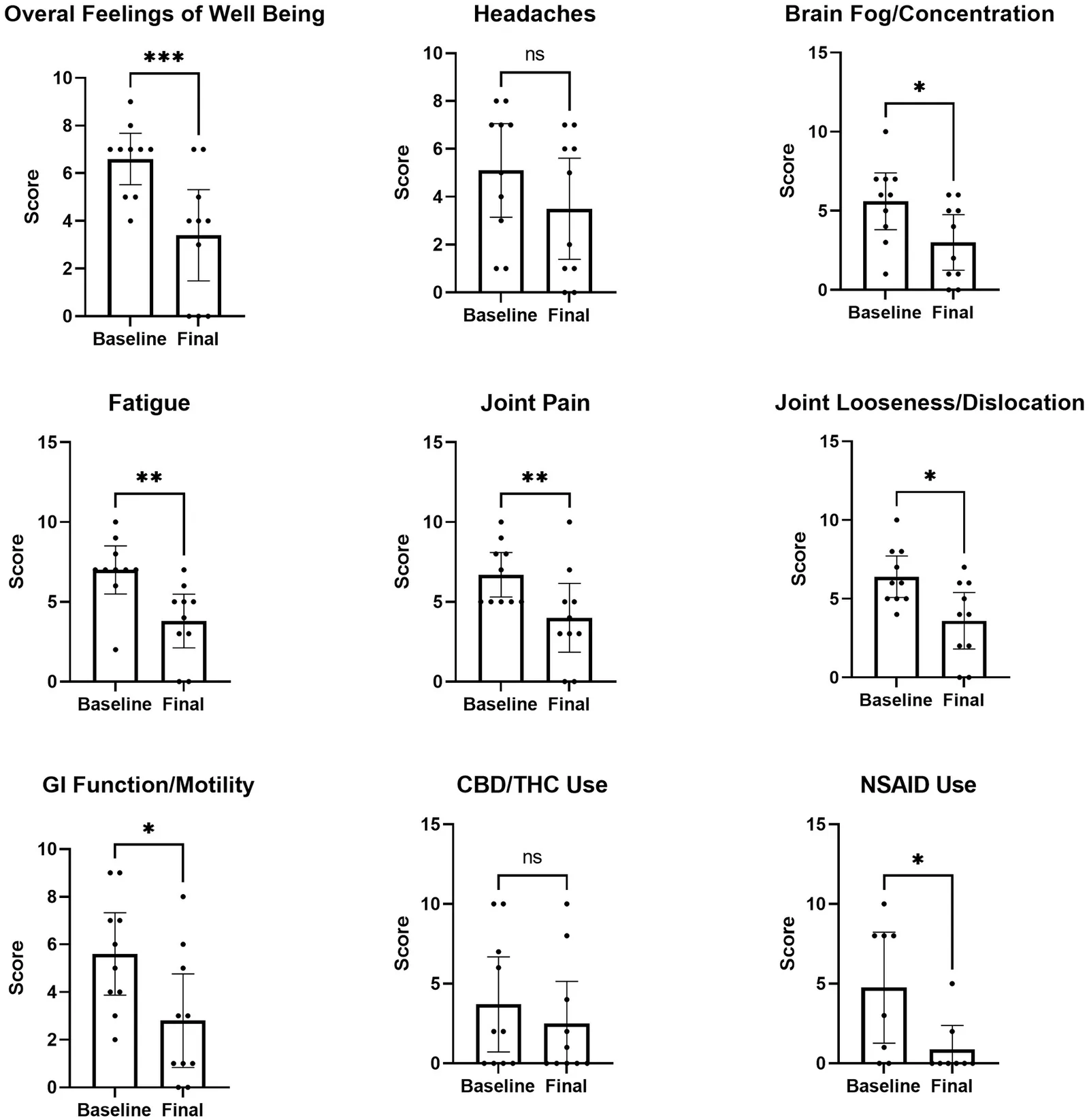
The IP causes statistically significant responses in multiple symptom parameters. Plots are shown with symbols representing individual patients, box height indicating the mean and whiskers showing 95% confidence intervals. Baseline parameter scores captured by the questionnaire one to three days before the first infusion of IP are shown on the left of each plot for the nine parameters. Scores obtained from each patient's parameters after their final dose are shown on the right of each plot. Statistical analysis was performed by t-test where *p < 0.05, **p < 0.01, ***p < 0.001, ns: not significant.

#### Designation as responder or non-responder patients

Criteria were established to classify patients as those who responded quickly and substantially to the treatment (responders) as compared to those who failed to respond or responded minimally (non-responders). Results were acquired from the 9-parameter questionnaire at baseline (prior to treatment) and two weeks after the initial dosing. For patients 1–6 the initial dosage was a single dose and for patients 7–10 the initial dosage was a double back-to-back dose. A patient was considered a responder only if a 3-point or greater reduction occurred in each of at least four of the nine symptoms assessed by the questionnaire (see Figure 3 and Supplementary Figures S3, 4).

**Figure 3:**
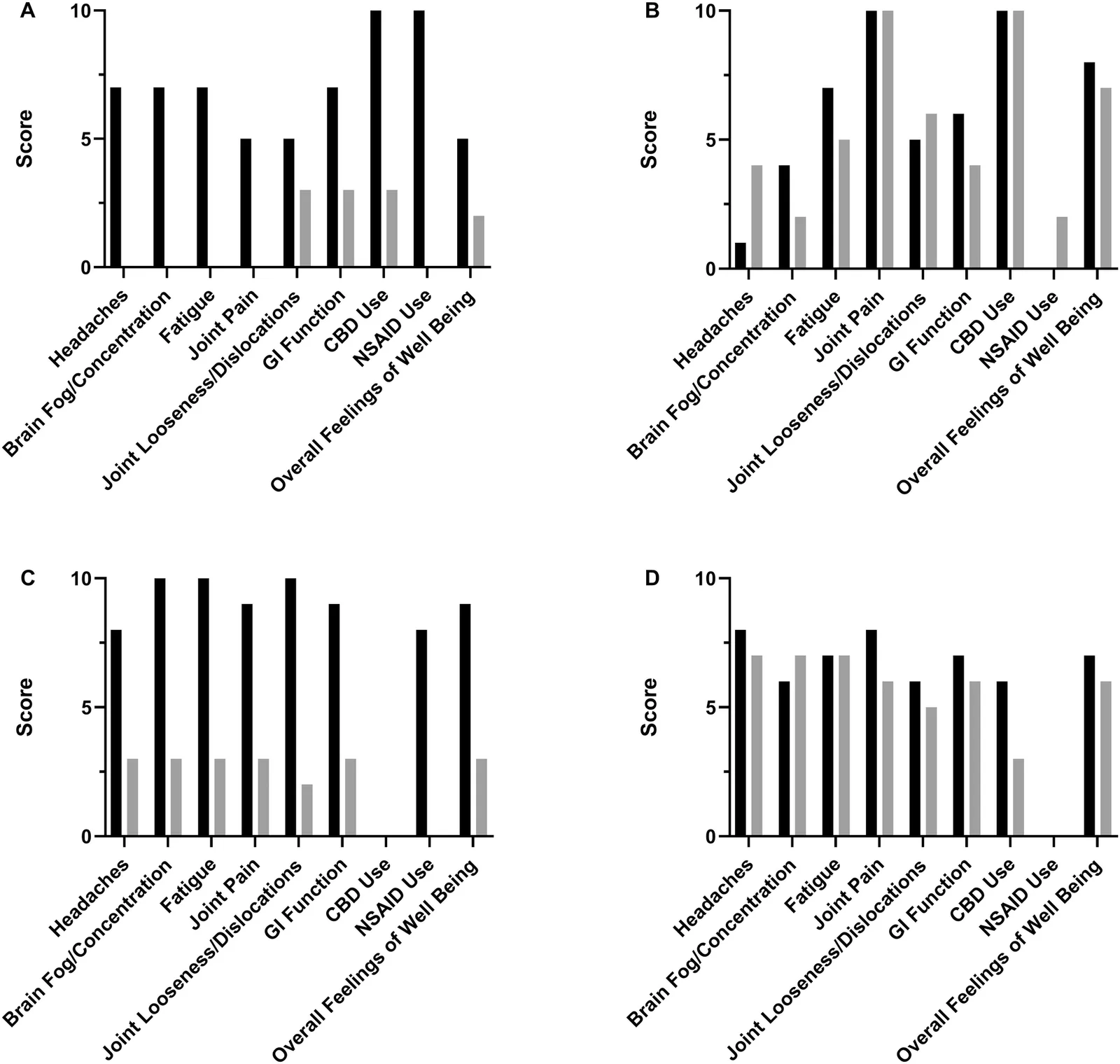
Examples of distinct, substantial, and rapid responders as well as non-responders. Individual symptom scores obtained at baseline (black) and at two weeks (gray) after the initial dose was administered. Panels **A** and **C** show responder patients. Panels **B** and **D** show non-responder patients.

#### Statistical analysis

The nine symptom questionnaire results were included in statistical analyses. Individual and summed score results for each patient were compiled and analyzed using GraphPad Prism 9.5.1 (GraphPad Software). Baseline scores were obtained for all patients one to three days prior to receiving the first infusion of IP. Scores were also obtained one to three days prior to each subsequent injection. All score results were compared to baseline scores using a paired t-test assuming a Gaussian distribution. A simple linear regression analysis was performed with Prism on the number of total doses received versus the percent reduction of each patient's summed scores to determine if there was a significant correlation between the total number of doses received and the patients' response to the IP. The percent change in summed score for each patient was calculated as [(baseline summed score – final summed score)/baseline]x100)).

## Results

### Safety

No adverse events of any grade or requiring medical intervention were reported or detected in any patient with these dose regimens. The dosing schedule for all patients, which was adjusted partway through the study, is summarized in Supplementary Information (Supplementary Figure S1). One patient failed to return for their final infusion. For another patient it was not possible to perform the final infusion due to difficulty finding a suitable vein that day. Recorded parameters available for these two patients were subjected to statistical analysis along with those of all others in the study.

### Efficacy

To address the possibility that patients might respond differently across symptoms to the IP, the scores from each assessment symptom after each dose regimen were summed to give an overall summed score for each patient. By analyzing the summed baseline scores at baseline and after the second dose for each of the 10 patients, a statistically significant response (*p* < 0.01) was observed for the patient group as a whole (Figure 1A). Some individuals (patients 5–8) showed a partial, unsustained or non response (Figure 1B). Thus, four (4) of the ten (10) patients were deemed non-responsive according to the criteria described in Methods. In contrast, the remaining six (6) patients (60%) showed rapid, distinct and sustained favorable responses. Both of the HLAB-27 positive patients (1, 9) were responders. No response was seen in the one patient (6) with a MTHFR (methylenetetrahydrofolate reductase) mutation. And no response was seen in either the patient (8) with a high positive ANA or the patient (5) who had previously received a cord blood stem cell infusion.

Response heterogeneity was further revealed by statistically analyzing and plotting results for each parameter obtained prior to treatment (baseline) and after each patient's final dose (Figure 2). Only the symptoms “Headaches” and “CBD/THC Use” failed to achieve statistical significance while all other symptom parameters achieved statistical significance throughout the study protocol. Individual patient responses for each parameter from this analysis are illustrated in Supplementary Figure S2. These results show that the IP elicited improvement across multiple symptoms in the patient group.

Figure 3 shows examples of response profiles from responder and non-responder patients two weeks after their initial dosage. Example results from two responders (Figures 3A,C) indicate that improvements occurred in all criteria for these patients. The non-responders (Figures 3B,D) were non-responsive or weakly responsive across all the symptom categories. Plots of individual symptom responses for all patients are shown in Supplementary Information (Figures 3, 4).

**Figure 4:**
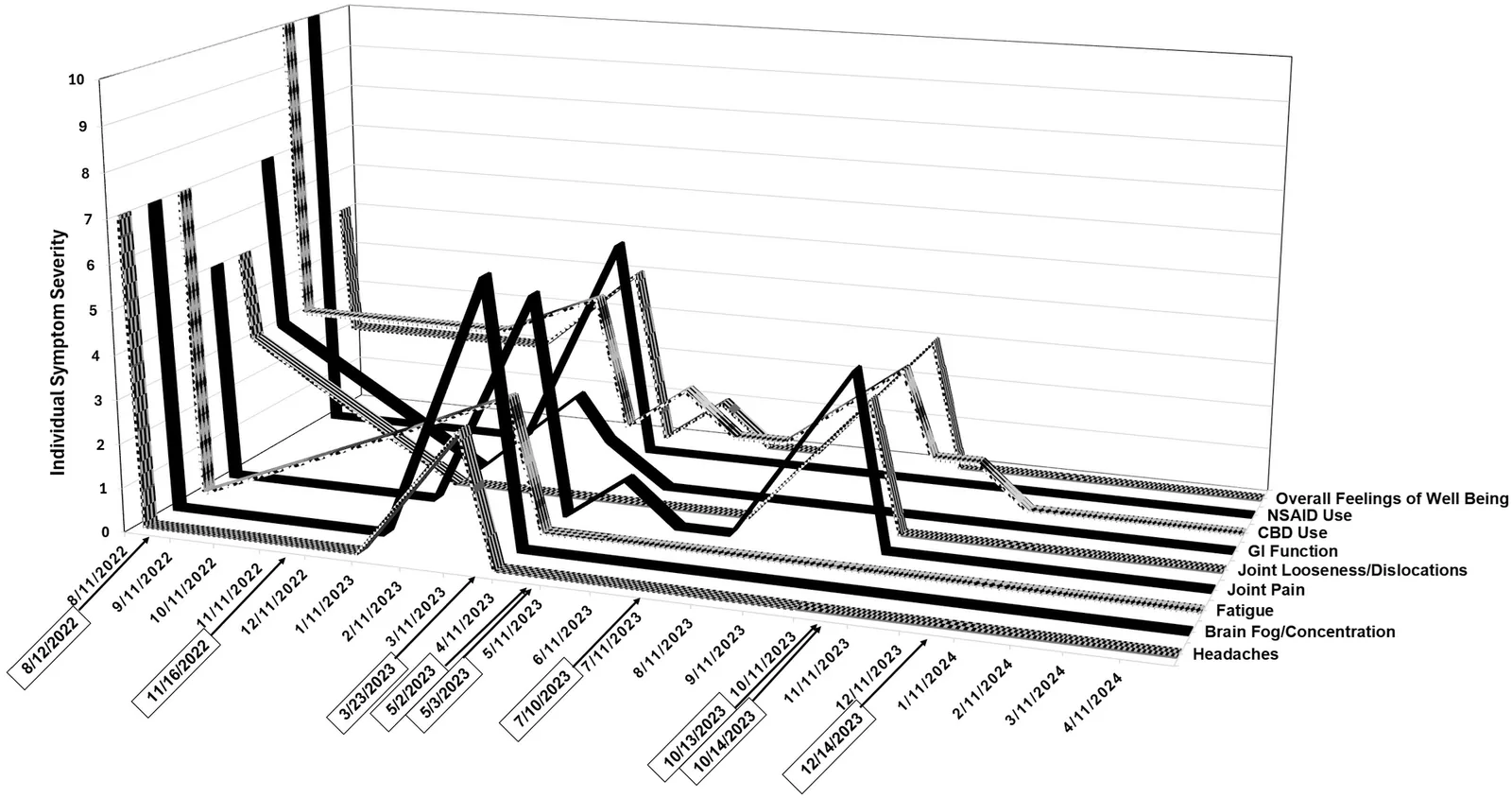
Only extended single patient symptom severity score for each symptom over 20 months after multiple additional infusions. Dosing schedule and treatment response for each assessed symptom parameter is shown. Independent scores for nine (9) symptoms reported on a given date were used to compile this graph. Arrows and dates within text boxes indicate dates of all nine infusions with 24-hour back-to-back double doses indicated on 5/2/23–5/3/23 and on 10/13/23–10/14/23.

In the six (6) distinctly responsive patients, notably in the parameter “Over-all Feelings of Well-being”, these patients showed substantial, rapid and sustained improvement. Responses to this query were also accompanied by patient remarks such as: “Much improved nightly sleep.’, “Drastic difference in symptoms..’, “Good experience in the clinical trial..’, “I am where I want to be physically..’, “I feel better than I have in years..’, “ It feels like I have hours back in my day..’, “…better than I ever thought I could be..’, “…improved outlook on life..’ and “…a miracle..’.

### Extended patient study

The extended patient's history and therapies used at the beginning of the entire study protocol are described in Tables 2, 3. The extended study patient was treated for a total of 16 months with 9 total infusions. Questionnaire results for each parameter for this patient only throughout 16 months of dosing (9 doses) and 4 months of follow-up are shown in Figure 4. Treatment was ultimately stopped at the patient's request and for nearly three years thereafter has lived without pain or any other of the previous symptoms or signs of Hypermobile Ehlers-Danlos Syndrome. All five (5) prior distinct responders with discontinued treatment reverted to their pre-treatment condition.

All parameters declined in severity after the first single dose, with “Brain Fog”, “Headache/Concentration”, “Joint Pain and NSAID Use” showing the greatest and most sustained improvement for this patient. A second dose was administered (11/16/2022) in an effort to improve other symptoms. Symptoms began to reappear within five months, and a third dose was provided (3/23/2023) leading to sustained improvement in the same four symptoms. To improve the remaining symptoms, two doses were administered within another two months (5/2/2023 and 5/3/2023). This led to lasting improvement in “GI Function”, but “CBD Use” remained elevated. A sixth dose (7/10/2023) was administered without reducing “CBD Use”. A worsening of “Joint Pain”, “Joint Looseness”, “CBD Use” and “Overall Well Being” symptoms in October of 2023 led to an additional back-to-back dosing (within 24 h), and all remaining symptoms except for “CBD Use” improved rapidly and completely. A final single dose was administered (12/14/2023) that was followed by rapid and sustained elimination of all recorded symptoms. At this writing, 32 months since the ninth and final dose, the patient has remained in apparent remission and not reported the recurrence of any of the symptoms measured here.

To evaluate the relationship between the total number of doses received and all patients' final response to the IP, a simple linear regression analysis was performed on a plot of each patient's percent improvement between their baseline summed scores and the summed score following their final dose (Figure 5). While a trend for increasing improvement with increasing number of doses was seen (slope of 11.33), the trend was not statistically significant at 95% confidence (*p* = 0.088).

**Figure 5:**
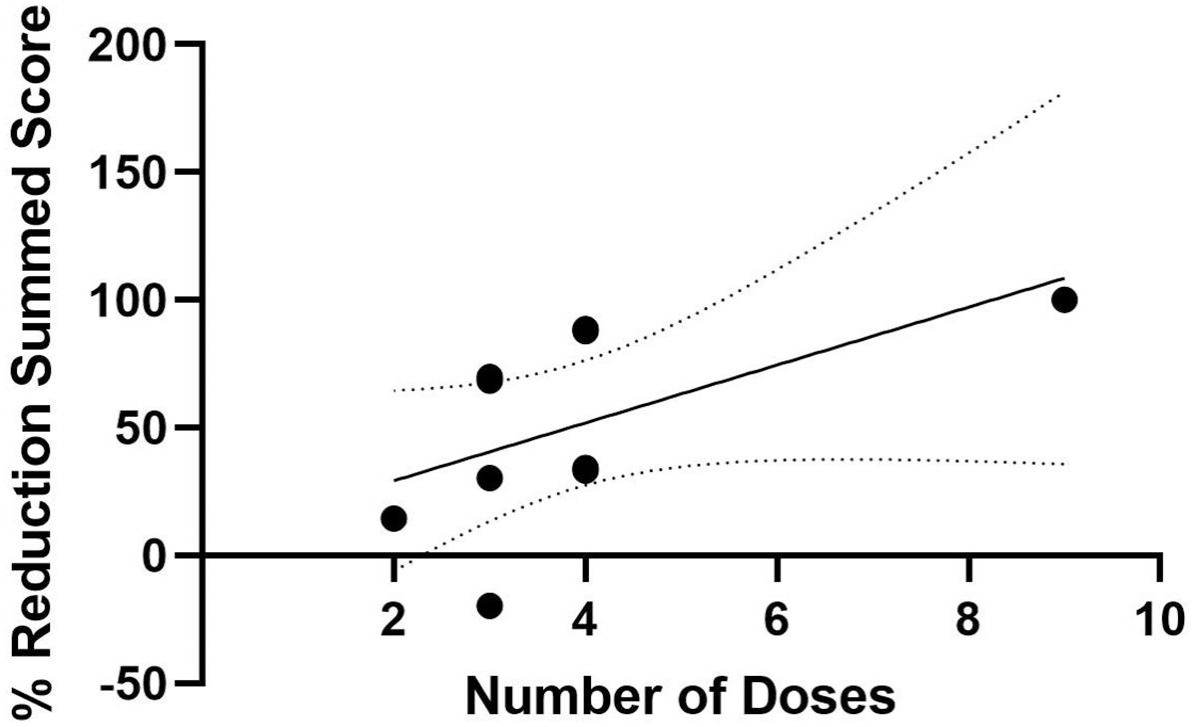
Simple linear regression analysis of summed scores determined two weeks after final doses for all 10 patients. Three patient values overlap with other patient values such that it appears that only seven patients are represented. Slope of the linear correlation line (solid) is 11.33 and the *p* value is 0.088. 95% Confidence Intervals are shown as dotted curves.

## Discussion

The IP (DB2Q) used in this study is currently in Phase 3 clinical trials for Acute Respiratory Distress Syndrome (ARDS, NCT05354141) (35). Based on our hypothesis that the properties of the MSC secretome could safely and favorably impact symptoms of hEDS, we sought to evaluate the safety of the IP in the patient population and the effect of the IP on the symptoms reported by a group of patients with hEDS. Results from an Expanded Access trial (NCT04657458) documenting the favorable safety profile of the IP have recently been summarized (41). No severe product-related adverse events have been noted across more than 300 patients. No treatment-related adverse events of any grade were reported here by the patients or noted by the investigators, which further extends the safety profile.

In this study of ten patients a single IV dose yielded distinct, substantial and rapid symptom relief that lasted approximately 10 weeks (Figure 2), which accounted for the 2-month dosage interval that was eventually adopted. All patients were examined personally by the PI before each infusion and either in office or by phone after each infusion. No clear advantage was observed in any patient after the double, back-to-back doses that were administered in an effort to improve responses as compared to single doses. At the end of the dosing regimen, the study group overall exhibited statistically significant responses in seven of the nine parameters assessed (Figures 1, 2). From these results we conclude that the IP safely, effectively and favorably modified multiple symptoms in this sample of hEDS patients. These preliminary signals of efficacy suggest that the IP has the potential to safely alleviate hEDS symptoms, a possibility that requires further evaluation in randomized, controlled clinical trials.

Within one to two weeks of the first infusion, six out of the 10 patients (60%) self-reported distinct, rapid and sustained favorable responses with a substantial reduction in symptoms that were sustained for the dosing period. These patients are considered responders to hbm-MSC EVs. Both of the HLAB-27 + patients were responders. In contrast, by closely inspecting the summed scores for each of the 10 patients, regardless of the dosing regimen, we conclude that one patient failed to respond favorably at all to the IP. Three patients showed a partial or unsustained response. Thus, four (4) of the ten (10) patients were determined non-responders. No response was seen in the one patient with a MTHFR (methylenetetrahydrofolate reductase) mutation. Likewise, no response was seen in the patient with a high positive ANA, which needs to be investigated further to determine if there is a mechanistic reason. The patient (5) who had previously received a dose of placental-derived cord blood stem cells was considered non-responsive to this treatment, and it is unclear if the previous treatment affected their response to the IP.

Relatively specific criteria for the diagnosis of hEDS have been proposed (2, 4, 15). The condition is subsumed under the heading of HSD which encompasses a broad group of hypermobile joint conditions (43). The usual first clue to hEDS, however, is the presence of painful, hypermobile joints. But not all hypermobile joints are painful and rheumatology evaluation and genetics consultation are usually in order. In this study we observed significant reductions in both joint pain and NSAID scores as reported using a 10-point scale. And, while all patients in this study were advised to continue any currently utilized pain reduction modalities and medications during the study, the responsive patients voluntarily either reduced or discontinued use of these as the study continued.

Based upon this pilot study, additional dosing may lead to a more favorable status. This proposal is supported by four observations. First, in the extensive weekly assessment scores, not all of which were included in our statistical analyses, for those that did respond to the first dose, symptoms began to return by 10 to 12 weeks post infusion. This prompted an adjustment in the protocol to infuse patients every two months rather than every four months with the goal of maintaining symptoms in check. We observed that some cumulative beneficial effects occurred in most responders with each of the subsequent infusions. Second, for the five responders who did not receive extended dosing, symptoms eventually returned. Third, the patient who received extended dosing has continued to show the same degree of improvement as was assessed within one week of their final dose. Fourth, while not statistically significant, the analysis of the patients' final summed scores showed a positive trend towards improved responses with increasing total doses. The favorable outcome of the extended dosing regimen for the extended dosing patient raises the possibility that the IP can contribute to recovery from disease symptoms for several years in the responsive patient population. Notably, 32 months after final infusion this patient is walking 3 to 4 miles every other morning without pain and without other symptoms related to his previously diagnosed hEDS. This level of activity was not possible prior to treatment. All current standard of care treatments for hEDS are only palliative.

The fact that some patients were non-responsive or only moderately responsive could be due to many reasons beyond the scope of this study. But this is consistent with the heterogenous character and lack of a defined single genetic basis for the disease (4, 15). Perhaps there are patients with phenotypical hEDS with differing and varied genotypes. Upon noting laboratory histories of the patients, we could not determine any definitive clinical or laboratory readings to explain differences between responders and non-responders.

The study had several limitations. It was not a randomized, placebo-controlled clinical study and confounding variables beyond the control of the investigators and participants may have influenced the results. As an investigator-initiated study it was small in scope and resources, which prevented acquisition of biomarkers and other relevant information. It was limited to ten patients, which in turn constrained the power of statistical analyses. A cross-over trial design to improve control of unconscious bias was not implemented since the persistence of potential effects of a regenerative medicine modality like the IP was unclear at the initiation of the study. The dosing regimens were adapted part way through the study in response to patient outcomes observed amongst the initial patients. So, the dosing regimen was not completely consistent throughout the study. Although clinical observation was conducted at every inquiry of the patients, the assessment of preliminary signals of efficacy was performed with a novel questionnaire developed specifically for hEDS symptoms and this has not been validated. Despite these limitations a statistically significant favorable response to the IP was observed amongst the group of 10 patients across seven of nine symptoms queried and across a summed score of the nine symptoms. Perhaps most interestingly, it appears that a truly sustained response could occur between 4 and 9 doses in a responsive patient. Based upon our clinical results, we cannot be more specific regarding optimal dosing duration. An expanded, randomized and controlled study across a larger number of patients will evaluate these and other possibilities.

The immunomodulatory capacity of the IP may contribute to improved regulation of the MCAS that is implicated in hEDS (16, 17). Additionally, the proinflammatory matrix-degrading phenotype typical of myofibroblasts that underlies distinct expression patterns of fibronectin, type I collagen and tenascin in hEDS and Hypermobile Spectrum Disorder (HSD) patients may also be modulated by the IP (12). Consistent with an immunomodulatory mechanism hypothesis, a recent proteomic evaluation of serum obtained from 29 female hEDS patients and from 29 matched healthy controls, with pathway enrichment and gene ontology analyses complemented by cytokine array data, strongly supports a role of dysregulated immunity being associated with hEDS pathology (21). Surprisingly, with the exception of ECM1, no other structural proteins like fibronectin or collagen, were differentially expressed. Instead, the majority of differentially expressed proteins were involved in immune responses (e.g., complement proteins, innate immunity), coagulation, blood pressure control and inflammation. The IP is highly enriched in proteins related to the complement cascade. Amongst the differentially expressed proteins identified in the proteomics paper, sixteen are downregulated in hEDS and thirteen of these have been identified in the IP. Thus, it is possible that the IP augments some of these downregulated proteins. These possibilities are suggested by inference from existing literature and further studies that include activity measures of pathways and associated biomarkers are needed to confirm such hypotheses. Other mechanisms, such as redox regulation, suggested by transcriptomic analyses, may also be affected by the IP (20). The efficacious activity and biochemical makeup of the IP, in light of these additional studies, indicate novel disease pathways may exist, which need to be investigated and perhaps targeted in the search for treatments for hEDS.

Hypermobile EDS (hEDS) is not a congenital condition in the sense that it can be diagnosed in early childhood. Except, perhaps, for hypermobile joints, the young child may appear normal. No specific clinical finding or laboratory test is diagnostic, although family history may be positive. Usually between 10 and 12 years old there may be complaints of foot, neck, jaw and other joint pain. A middle-school child may have trouble keeping up with classmates at gym or on the field. Not uncommonly, the child is accused by teachers and coaches of laziness and lack of toughness. Self-esteem and self-doubt issues may arise. Parents are frequently unaware of issues at school and only vaguely aware of issues at home. Once addressed as a possible medical issue, misdiagnosis over several years is common. Multiple doctors are usually consulted before a confident diagnosis is proposed. The day of diagnosis is often declared as the “best day of my life” by the late-teenage patients. Many are diagnosed in their mid-twenties and beyond. The apparent safety of the IP and, as suggested in this study, potential for efficacy across multiple symptoms indicate that more rigorous clinical studies may lead to approval of a highly sought-after new biologic entity for treatment of hEDS. Additional use of this biologic drug candidate and increased understanding of its mechanism of action may also reveal new pathophysiological avenues towards improving both diagnosis and treatment of this otherwise intractable disease.

## Data Availability

The raw data supporting the conclusions of this article will be made available by the authors, without undue reservation.
