# Supplementary material for: A trial of exosome rich stem cell secretome as a potential treatment for Hypermobile Ehlers-Danlos Syndrome
Source: Front Med (Lausanne). 2026 Sep 29;13:1945865. doi: 10.3389/fmed.2026.1945865 (PMC13657981; doi:10.3389/fmed.2026.1945865)
Supplement: Supplementary file 1 [file Datasheet1.docx]

**SUPPLEMENTAL INFORMATION**

SI Figure 1. Dosing schedule of the study. Patients are listed in the left column. Dosing and assessment periods (1 – 9) are indicated across the second row. Summed score responses for each patient before each dose (pre-) and two weeks after each dose (2 wk post-) are shown in gray highlighted columns. Back-to-back infusion notations indicate two doses were provided at 24-hour intervals and therefore span two dosing periods. The two patients who were unable to receive their final doses are indicated as “Unsuccessful infusion.”

SI Figure 2. Patient responses to IP treatment indicate the existence of responders and non-responders. Baseline scores (left in each plot) for the nine assessed parameters (panels A-I) are plotted for each patient and connected by a solid line to their final score (right in each plot) for the same patient. Some patients have identical start and/or ending scores so it may erroneously appear there are fewer patients.

SI Figure 3. Individual symptom scores obtained at baseline (black) and at two weeks (gray) after the initial single dose for the first six patients. Patients 5 and 6 were determined to be non-responders as described in Protocol.

SI Figure 4. Individual symptom scores obtained at baseline (black) and at two weeks (gray) after the initial back-to-back double dose for the remaining four patients. Patients 7 and 8 were determined to be non-responders as described in Protocol.
